# Gene expression profile of human follicle dermal papilla cells in response to *Camellia japonica* phytoplacenta extract

**DOI:** 10.1002/2211-5463.13076

**Published:** 2021-02-14

**Authors:** Won Kyong Cho, Hye‐In Kim, Seung Hye Paek, Soo‐Yun Kim, Hyo Hyun Seo, Jihyeok Song, Ok Hwa Lee, Jiae Min, Sang Jun Lee, Yeonhwa Jo, Hoseong Choi, Jeong Hun Lee, Sang Hyun Moh

**Affiliations:** ^1^ Research Institute of Agriculture and Life Sciences College of Agriculture and Life Sciences Seoul National University Korea; ^2^ Anti‐aging Research Institute of BIO‐FD&C Co., Ltd. Incheon Korea

**Keywords:** callus, *Camellia japonica*, hair follicle, placenta extract, RNA‐seq, transcriptome

## Abstract

*Camellia japonica* L. is a flowering tree with several medicinal and cosmetic applications. Here, we investigated the efficacy of *C. japonica* placenta extract (CJPE) as a potential therapeutic agent for promotion of hair growth and scalp health by using various *in vitro* and *in vivo* assays. Moreover, we performed transcriptome analysis to examine the relative expression of human follicle dermal papilla cells (HFDPC) in response to CJPE by RNA‐sequencing (RNA‐seq). *In vitro* assays revealed upregulation of the expression of hair growth marker genes in HFDPC after CJPE treatment. Moreover, *in vivo* clinical tests with 42 adult female participants showed that a solution containing 0.5% CJPE increased the moisture content of the scalp and decreased the scalp's sebum content, dead scalp keratin, and erythema. Furthermore, RNA‐seq analysis revealed key genes in HFDPC which are associated with CJPE. Interestingly, genes associated with lipid metabolism and cholesterol efflux were upregulated. Genes upregulated by CJPE are associated with several hormones, including parathyroid, adrenocorticotropic hormone, α‐melanocyte‐stimulating hormone (alpha‐MSH), and norepinephrine, which are involved in hair follicle biology. Furthermore, some upregulated genes are associated with the regulation of axon guidance. In contrast, many genes downregulated by CJPE are associated with structural components of the cytoskeleton. In addition, CJPE suppressed genes associated with muscle structure and development. Taken together, this study provides extensive evidence that CJPE may have potential as a therapeutic agent for scalp treatment and hair growth promotion.

## Graphical abstract

Here, we examined the efficacy of *C. japonica* placenta extract (CJPE) as a potential therapeutic agent for promotion of hair growth and scalp health by using various *in vitro* and *in vivo* assays. Moreover, we conducted transcriptome analysis to examine the relative expression of human follicle dermal papilla cells in response to CJPE by RNA‐sequencing.

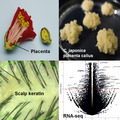

**Abbreviations**
CJPE: *Camellia japonica* phytoplacenta extract
DEG: differentially expressed genes
HaCaT: human immortalized keratinocyte
HFDPC: human follicle dermal papilla cells
RNA‐seq: RNA‐sequencing

*Camellia japonica* is a flowering tree native to Korea, China, Taiwan, and Japan. In Korea, *C. japonica* can be easily found on several islands, including Jeju and Ulleung Islands. *C. japonica* belongs to the genus *Camellia* in the family Theaceae, and it is known as common camellia, Japanese camellia, rose of winter, and *Dongbaek* in Korean [1]. With its beautiful flowers, *C. japonica* is an important ornamental species. To date, a large number of *C. japonica* cultivars have been developed [2].

For a long time, extracts of camellia flowers and oil from camellia seeds have been used as traditional medicines and cosmetics in several Asian countries. Moreover, many previous studies have demonstrated biological effects of camellia extracts from various tissues as medicines. For example, extracts from petals and leaves of the camellia flower show antibacterial activity against foodborne pathogens [3, 4]. Similarly, extracts from leaves and flowers of camellia have antifungal and antioxidant activities [5, 6]. In addition, camellia leaf extracts have anti‐allergic [7, 8], anti‐photoaging [9], and antiatherogenic activities [10]. Furthermore, extracts of camellia can be used as cosmetic materials [11, 12]. For instance, a recent study showed skin protective effects of flower extracts of camellia against urban air pollutants due to its antioxidant activity [13].

Several important biological components have been isolated from camellia. Of them, two triterpenoid saponins have been isolated from camellia leaf extracts [14]. Furthermore, camellia pericarp extracts containing camelliatannin H showed strong inhibitory effects against human immunodeficiency virus (HIV) [15]. Moreover, a study extracted red pigments from camellia and isolated (−)‐epicatechin as an active compound exhibiting excellent 1,1‐diphenyl‐2‐picrylhydrazyl radical‐free radical scavenging activities [16].

In flowering plants, the placenta, known as the phytoplacenta, is the surface of the carpel to which the ovules are attached. The placenta connects the ovary walls and provides nutrition to the developing ovules. Although the biological activities of camellia extracts from different tissues, such as leaf, seed, flower, and fruit tissues, have been well reported, the biological activities of *C. japonica* phytoplacenta extract (CJPE) have not been well characterized.

A plant callus is an unorganized or undifferentiated cell mass that can be easily generated by wounding the plant and can be artificially cultured in antiseptic growth conditions. Plant callus cells have totipotency and plasticity like animal stem cells, since plant callus cells can develop into all the various cell types of an organism [17]. With a plant‐specific bioreactor, it is possible to generate a large quantity of specific plant cells within a short time. Thus, without mature plant cultivation, using the plant callus with a plant‐specific bioreactor facilitates the production of diverse plant extracts with a quality equal to that of mature plants [18].

Human hair has a wide range of functions. For example, hair has long been regarded as an important factor in human attraction, and it can provide physical protection against ultraviolet rays from the sun as well as hot and cold temperatures [19]. The human hair follicle is an organ residing in the dermal layer of the skin composed of 20 different cell types [20]. In the epithelial bulge region, hair follicle stem cells regenerate the hair follicle during cycling [21]. The dermal compartment of the hair follicle is composed of the dermal papilla and dermal sheath, and the interaction between epithelial and dermal papilla cells plays a main role in hair follicle morphogenesis [21]. In addition, several molecular mechanisms regulating hair follicle development have been identified [22].

In this study, we investigated the efficacy of CJPE derived from *C. japonica* placenta as a therapeutic agent for the improvement of hair growth and scalp health by various *in vitro* and *in vivo* assays. In addition, we carried out transcriptome analysis to examine the relative expression of human follicle dermal papilla cells (HFDPC) in response to CJPE by RNA‐sequencing (RNA‐seq).

## Materials and methods

### Production of callus from *C. japonica* placenta

Flowers of *C. japonica* were obtained from the Jeju Plant Resources Institute on Jeju Island, Korea. The Jeju Plant Resources Institute carried out the formal identification of the plant material (*C. japonica*) used in this study. The plant material of *C. japonica* is available from the Jeju Plant Resources Institute, which collects diverse naturalized plants on Jeju Island (http://www.jpri.co.kr/index.php). To obtain the placenta tissue of *C. japonica*, the bottoms of flowers were cross‐sectioned using a pincette and a mess. The *C. japonica* placenta tissue was cut into small pieces (0.5–1 cm) and then sterilized by soaking in 70% EtOH for 30 s followed by washing with distilled water. After that, the *C. japonica* placenta tissue was shaken in 0.3% sodium hypochlorite (Waco, Osaka, Japan) for 20 min and washed with distilled water. The early stage of plant cells was induced on Murashige and Skoog (MS) medium containing 0.5–3 mg·mL^−1^ of 6‐benzylaminopurine (Duchefa Biochemie, Haarlem, Netherlands) and 0.3–1 mg·mL^−1^ of 2,4‐dichlorophenoxyacetic acid (2,4‐D) (Duchefa Biochemie) in darkness at 25 ± 2 °C [15]. The pH of the MS medium was adjusted to 5.8 using 1N of NaOH (Duchefa Biochemie). The induced callus was propagated in a petri dish, after which the selected callus line was cultured in a bioreactor at the Anti‐Aging Research Institute of BIO‐FD&C Co., Ltd., Incheon, Korea. The cultured callus was harvested and washed three times with distilled water and then dehydrated using a freeze drier (IlShinBioBase, Dongducheon‐si, Korea) according to the manufacturer's instructions. The dried camellia callus was stirred in distilled water at 50 °C for 8 h. We subsequently extracted CJPE by heat extraction with 10 g of *C. japonica* callus in 1 L of distilled water at 98 °C for 1 h.

### Cultivation of human cells

Human immortalized keratinocyte (HaCaT) cells (ATCC, Manassas, VA, USA) were cultivated in Dulbecco's modified Eagle medium (Welgene, Gyeongsan, Korea) supplemented with 10% FBS (Thermo Fisher Scientific, Waltham, MA, USA) and 1× antibiotic–antimycotic solution (Thermo Fisher Scientific) at 37 °C in 5% CO_2_. HFDPC isolated from mainly normal human scalp hair follicles was purchased from ScienCell (San Diego, USA). It was cultivated in mesenchymal stem cell medium‐basal (MSCM‐b) (ScienCell) including 10% FBS and 1× antibiotic solution at 37 °C in 5% CO_2_. In this study, eight and six passages were performed for HaCaT and HFDPC, respectively.

### Expression analysis by real‐time reverse transcription (RT)‐PCR

For the evaluation of moisturizing by CJPE treatment, HaCaT cells at a density of 5 × 10^4^ cells per well were incubated with CJPE and sterile water separately in a 96‐well plate for 24 h. HFDPC was used to evaluate the effects of CJPE on hair follicle cell growth. HaCaT cells and HFDPC were treated with three different concentrations of CJPE (0.5%, 1%, and 2%) and minoxidil (Sigma‐Aldrich, St. Louis, MO, USA) as well as sterile water as a control separately. After treatment, we extracted total RNA from each sample using an RNeasy Mini Kit (Qiagen, Hilden, Germany) according to the manufacturer's instructions. We synthesized cDNA from the extracted total RNA using a SuperPrep Cell Lysis & RT Kit for qPCR (Toyobo, Osaka, Japan) containing lysis reagents and RT reagents according to the manufacturer's instructions. For real‐time RT‐PCR, we used a known primer for *Aquaporin 3* (*AQP3*) (Product number: QT00212996) as a moisturizing marker. We also used known primers for *β‐Catenin* (*CTNNB1*) (Product number: QT00057134), *keratinocyte growth factor* (*KGF*) (Product number: QT01194676), *vascular endothelial growth factor* (*VEGF*) (Product number: QT00013783), *versican* (*VCAN*) (Product number: QT00064064), *alpha‐smooth muscle actin* (*ACTA2*) (Product number: QT00088102), and *alkaline phosphatase* (*ALPL*) (Product number: QT00012957) as hair follicle growth markers (Qiagen). Real‐time RT‐PCR was conducted using a Thunderbird SYBR qPCR Mix Kit (Toyobo) based on the manufacturer's instructions.

### Ethics approval and consent to participate for *in vivo* clinical test of CJPE

The *in vivo* clinical test was conducted by KC Skin Research Center Co., Ltd. (Seoul, Korea) to evaluate the effect of CJPE on scalp moisturization, sebum content, keratin, and redness. This study was based on the test method guidelines (2018.03) of KC Skin Research Center Co., Ltd. after the IRB (KC‐180716‐C1) approval for the validation of cosmetics labels and advertisements at the Ministry of Food and Drug Safety and KC Skin Research Center Co., Ltd.'s Standard Operating Procedure. A total of 42 adult females aged 19–60 years participated: 21 participants used the test product, while 21 participants used the control product. Consequently, we used the results of 40 participants and excluded the results of two participants. The study was conducted according to the guidelines set by the declaration of Helsinki, and each subject signed an informed consent before participating to the study.

### Criteria for inclusion, exclusion, and withdrawal of subjects in clinical test

Subject inclusion criteria were as follows: healthy subjects aged 19–60 years, subjects who voluntarily signed the consent form after understanding the study's objective and contents, subjects who could be observed and traced during the study period, and subjects who did not meet any of the exclusion criteria.

Subject exclusion criteria were as follows: subjects who did not wish to apply or who did not sign the consent form; subjects who had dieted or taken medicine within the last 6 months; subjects who had smoked within the last 6 months or were currently smoking; subjects who had permanently dyed or bleached their hair within the last 6 months; subjects who had diathesis or boils, wounds, dermatitis, or eczema on the scalp, face, neck, or back of the hand; subjects who had participated in the same study within 3 months prior to the start of the study; subjects who were pregnant or lactating or were planning to become pregnant within 3 months; and subjects who were considered inappropriate for the study by the chief researcher or researcher in charge.

Subject withdrawal criteria were as follows: subjects who wished to discontinue study participation, subjects who could not proceed due to the occurrence of a skin disease during the study, subjects who had serious adverse reactions after using the test products, and subjects who violated the method of use or schedule without a valid reason.

### Measurement of scalp moisturization in response to CJPE treatment

Scalp moisturization was measured by a Corneometer (Courage + Khazaka Electronic, Köln, Germany) at three different time points: before (Time Point A), immediately after (Time Point B), and 1 week after (Time Point C) product use. The moisture content of the scalp area was measured three times, and the measured values were averaged for the evaluation of scalp moisturization. For the moisture content, we used an arbitrary unit, a proportional value of scalp moisturization.

### Measurement of scalp sebum in response to CJPE treatment

Scalp sebum was measured by a Sebumeter (Courage + Khazaka Electronic) at the same three different time points used for scalp moisturization. We measured the sebum content in the scalp area once. For the sebum content, we used μg·cm^−2^ as a unit, a proportional value of scalp sebum. In general, smaller values indicate improvements of the scalp sebum.

### Measurement of scalp keratin in response to CJPE treatment

Scalp keratin was measured using Corneofix (Courage + Khazaka Electronic), a special adhesive tape, at the same three different time points used for scalp moisturization. We removed the dead skin cells of the scalp and photographed them with a Visioscan VC98 (Courage + Khazaka Electronic). Using the photographs, we calculated the average dead skin cell index (DI: desquamation index), a proportional value of scalp keratin. We used the percentage as the unit of dead skin cells. In general, smaller values indicate improvements of scalp keratin.

### Measurement of scalp redness improvement in response to CJPE treatment

Scalp redness improvement was measured by a Mexameter (Courage + Khazaka Electronic) at the same three different time points used for scalp moisturization. The erythema dose of the scalp area was measured three times, and the measured values were averaged for the evaluation of scalp redness improvement. We used the erythema index as a unit, a proportional value of scalp erythema. In general, smaller values indicate improvements of scalp redness. Scalp images were taken by a folliscope 4.0 (LeadM, Seoul, Korea).

### Preparation of libraries for RNA‐seq

For the transcriptome analysis, HFDPC at a density of 1 × 10^6^ cells per well was incubated in a six‐well plate for 24 h. After that, HFDPC (treatment) was treated with a final concentration of 2% CJPE for 24 h, while the mock condition (control) was treated with sterile water. For each condition, three different biological samples were harvested. Total RNA was extracted using an RNeasy Mini Kit (Qiagen) according to the manufacturer's instructions. After the extraction of total RNA from each sample, six different libraries for RNA‐seq were prepared using the TruSeq Stranded mRNA LT Sample Prep Kit according to the manufacturer's instructions and then paired‐end sequenced by Illumina's NovaSeq 6000 system (Macrogen, Seoul, Korea). The obtained raw sequence data were deposited in the National Center for Biotechnology Information (NCBI) Sequence Read Archive (SRA) database with the respective accession numbers SRR11774226–SRR11774231 under the project PRJNA631804.

### Mapping, normalization, and identification of differentially expressed genes (DEGs)

We mapped the raw sequence reads from each library to human reference transcripts version GRCh38 (https://www.ncbi.nlm.nih.gov/genome/guide/human/) using the Burrows‐Wheeler Aligner [23] with default parameters (http://bio‐bwa.sourceforge.net/). Using the pileup.sh option implemented in BBMap (https://sourceforge.net/projects/bbmap/), we calculated the number of mapped reads for each transcript. The obtained read numbers from each condition were subjected to the Gene Expression Normalization Analysis and Visualization (GENAVi) online tool (https://junkdnalab.shinyapps.io/GENAVi/) for normalization [24]. Using the DESeq2 package implemented in GENAVi, differential expression analysis between treatment and control conditions was conducted. Finally, we identified DEGs according to *P*‐values of < 0.01 and log_2_ converted fold changes in more than 1.

### Gene ontology (GO) term enrichment analysis

To identify enriched functions for DEGs, the identified DEGs were divided into two groups: upregulated genes (161 transcripts) and downregulated genes (93 transcripts). The DEGs in the two different groups were subjected to the WEB‐based GEne SeT AnaLysis Toolkit (WebGestalt) (http://www.webgestalt.org/) for GO term enrichment analysis [25]. We identified enriched GO terms group according to biological from the GO database for each process (BP), cellular component (CC), and molecular function (MF). Significantly enriched GO terms in each group were visualized by a directed acyclic graph (DAG) structure generated by WebGestalt. We also identified overrepresented pathways for the DEGs against five different databases (KEGG, Panther, Reactome, WikiPathways, and WikiPathways Cancer) using WebGestalt.

### Statistical test

For comparisons within groups, we conducted paired‐samples *t*‐tests (*: *P* < 0.05, **: *P* < 0.01, ***: *P* < 0.001) and Wilcoxon signed‐rank tests (^†^: *P* < 0.05). For comparisons between groups, we conducted independent *t*‐tests (^#^: *P* < 0.05). All statistical tests were declared statistically significant at the 0.05 level. We used IBM spss Statistics version 21.0 (SPSS, Chicago, IL, USA) for the statistical analyses.

## Results

### Preparation of CJPE from *C. japonica* placenta

**Fig. 1:**
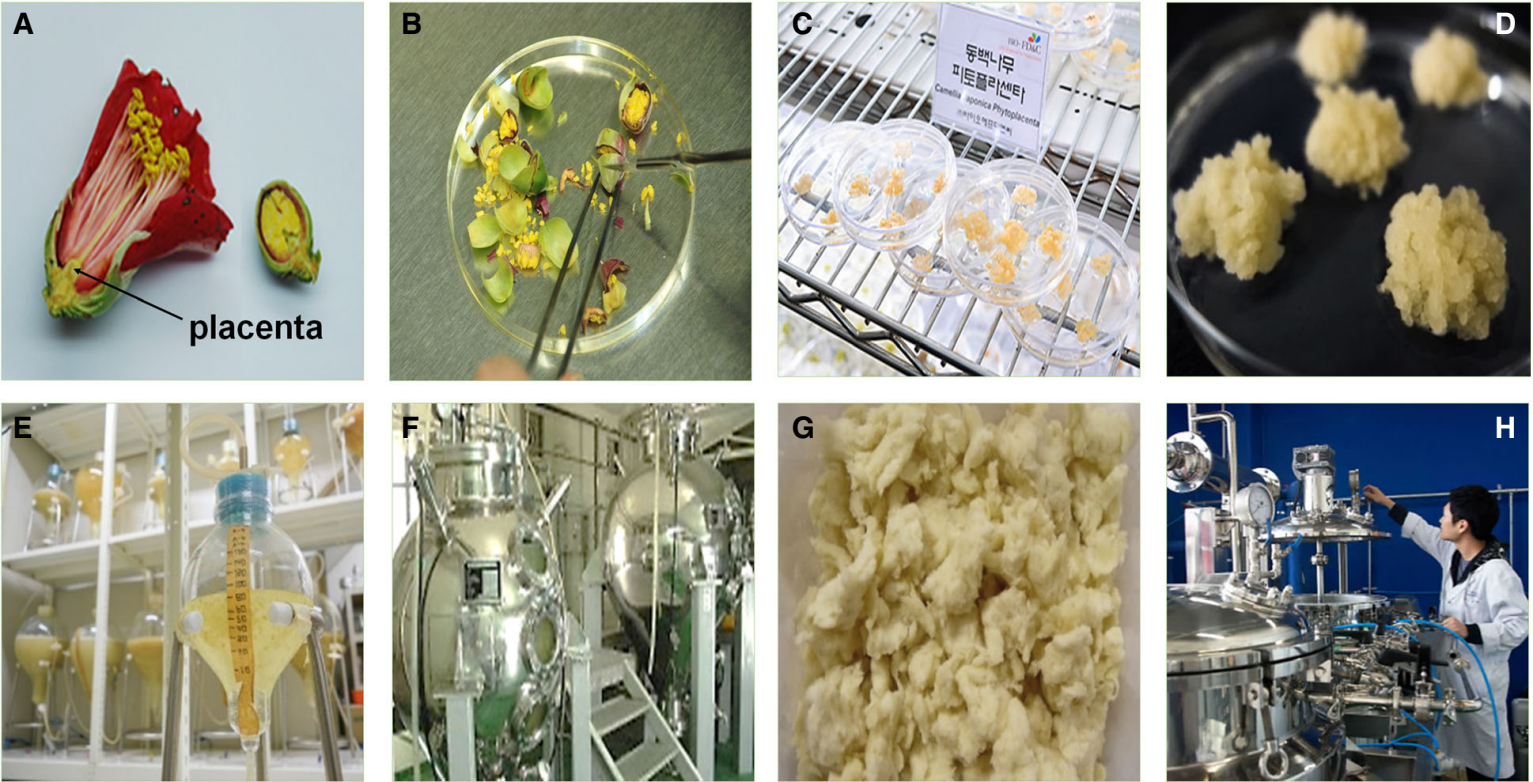
Experimental procedure to obtain CJPE from *C. japonica* placenta callus using plant bioreactors. (A) Cross section of *C. japonica* flower showing position of placenta (black arrow). (B) Selection of placenta tissues from *C. japonica* flowers. (C) Induction of callus from *C. japonica* placenta. (D) Magnification of *C. japonica* placenta callus. (E) Suspension culture of induced callus. (F) Cultivation of *C. japonica* placenta callus using bioreactors. (G) Harvest and lyophilization of *C. japonica* placenta callus. (H) Heat extraction of *C. japonica* placenta callus cells.

The placenta is located at the bottom of the flower, as shown in the cross section of *C. japonica* (Fig. 1A). Using a pincette and a mess, we selected the placenta tissue from *C. japonica* flowers (Fig. 1B). The placenta tissues were first sterilized and subjected to callus induction (Fig. 1C,D). The induced callus was used for the suspension cell culture (Fig. 1E). For the large‐scale production, *C. japonica* placenta calluses were cultured using bioreactors (Fig. 1F). Cultured callus cells were then harvested and lyophilized (Fig. 1G). We extracted CJPE by heat extraction (Fig. 1H). The different CJPE concentrations were used for multiple *in vitro* (0.5%, 1%, and 2%) and *in vivo* (0.5%) assays and transcriptome analysis (2%).

### *In vitro* evaluation of CJPE as a moisturizing agent

**Fig. 2:**
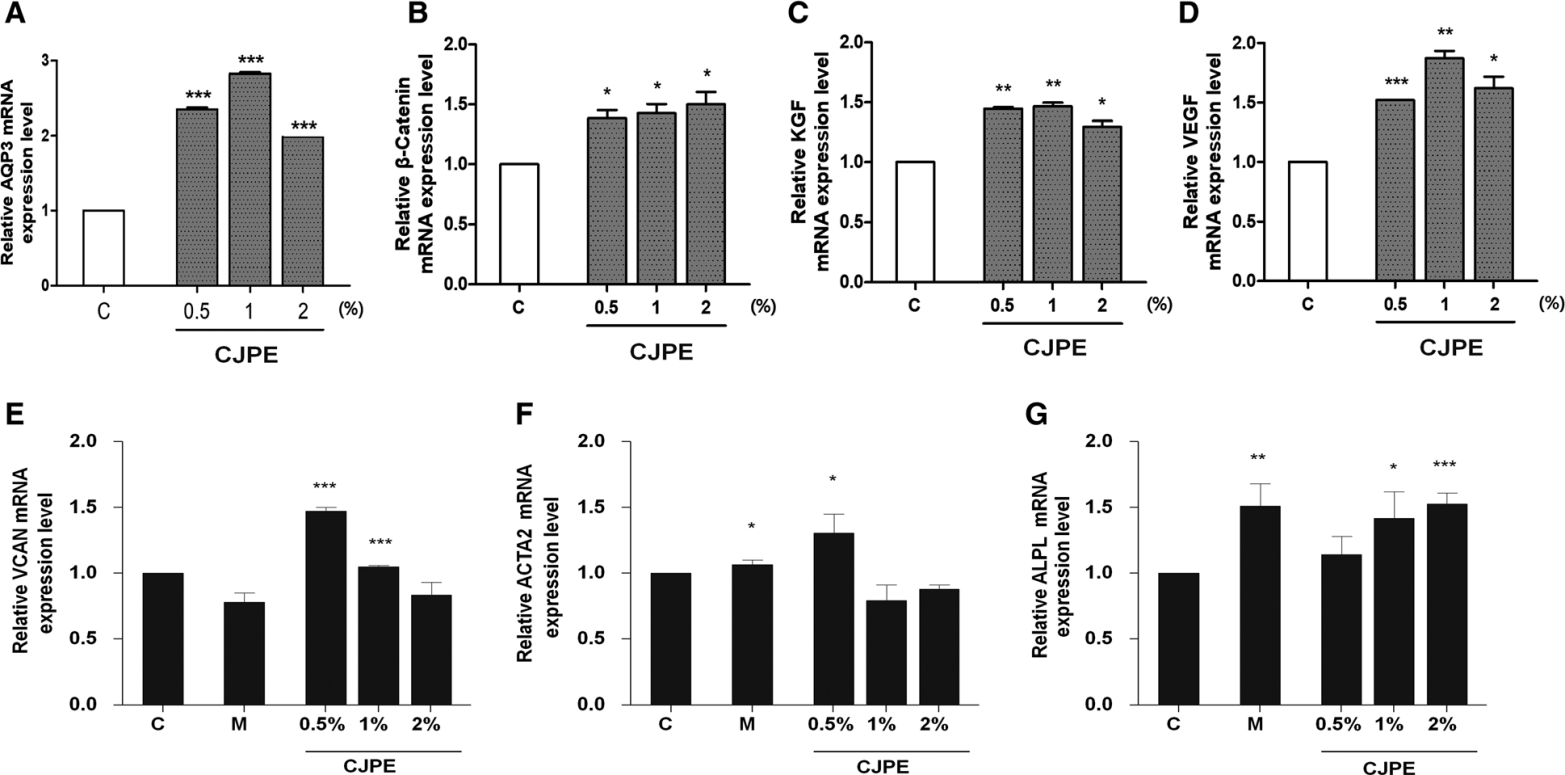
*In vivo* assessment of CJPE as moisturizing and hair follicle growth‐promoting agent by real‐time RT‐PCR. (A) Relative expression of *AQP3* in HaCaT cells in response to different CJPE concentrations. Relative expression of six hair growth marker genes encoding *CTNNB1* (B), *KGF* (C), *VEGF* (D), *VCAN* (E), *ACTA2* (F), and *ALPL* (G) in HFDPC in response to treatments with three different CJPE concentrations. C and M indicate control (distilled water) and minoxidil, respectively. For comparisons within groups, we conducted paired‐samples *t*‐tests. Statistical significance indicated by * (*P* < 0.05), ** (*P* < 0.01), and *** (*P* < 0.001). The error bars represent the SD of a data set. The *N* value (number of biologically independent replicates) was 3.

To examine the moisturizing effect of CJPE on HaCaT cells [26], we carried out real‐time RT‐PCR to investigate the expression of *AQP3*, which encodes a water‐ and glycerol‐transporting protein expressed in skin cells (Fig. 2A). The expression of *AQP3* was significantly increased by the CJPE treatment as compared to the control. Among the three different CJPE concentrations, the relative expression of *AQP3* was the highest in 1% CJPE (3‐fold change) followed by 0.5% (2.5‐fold change) and 2% CJPE (2‐fold change) (Fig. 2A).

### *In vitro* evaluation of CJPE for proliferation of HFDPC

In order to reveal the effects of CJPE on the proliferation of HFDPC, we examined the expression of three different hair follicle cell growth marker genes encoding *CTNNB1* [27], *KGF* [28], and *VEGF* [29] by real‐time RT‐PCR. HFDPC isolated from mainly normal human scalp hair follicles was treated by three different concentrations (0.5%, 1%, and 2%) of CJPE. As compared to the control (sterile water), the relative expression of *CTNNB1* in HFDPC was increased up to about 1.5 times by CJPE treatment regardless of concentration (Fig. 2B). In addition, as the CJPE concentration increased, the expression of *CTNNB1* increased slightly (Fig. 2B). Moreover, the expression of *KGF* and *VEGF* was induced by CJPE treatment (Fig. 2C,D). Among the three different CJPE concentrations, the 1% CJPE treatment resulted in the highest expression for both *KGF* and *VEGF* genes. Although the 2% CJPE increased the expression of the three hair follicle growth marker genes as compared to control, the expression of the three genes was not always the highest among the three different CJPE concentrations (Fig. 2B‐D). Currently, minoxidil is widely used for the treatment of male pattern hair loss [30]. We thought it would be of interest to determine whether minoxidil promotes the expression of genes associated with hair follicle growth. For that, HFDPC was treated by 1 µm minoxidil and three different concentrations of CJPE. We examined the expression of three additional hair follicle growth marker genes, VCF, α‐SMA, and ALP, by real‐time RT‐PCR (Fig. 2E–G). The expression of the *VCAN* gene was slightly reduced by the minoxidil treatment, while the 0.5% CJPE treatment significantly induced the expression of the *VCAN* gene (1.475‐fold change) (Fig. 2E). The expression of the *ACTA2* gene (1.305‐fold change) was highly induced by the 0.5% CJPE treatment, whereas the treatment with 1% and 2% CJPE slightly reduced the expression of the *ACTA2* gene (Fig. 2F). The expression of the *ACTA2* gene was not changed by the minoxidil treatment (Fig. 2F). The expression of the *ALP* gene (1.51‐fold change) was strongly upregulated by the minoxidil treatment (Fig. 2G). Moreover, the 1% and 2% CJPE treatments induced the expression of the *ALP* gene (Fig. 2G).

### *In vivo* clinical assessment of CJPE for hair follicle cell improvement

**Fig. 3:**
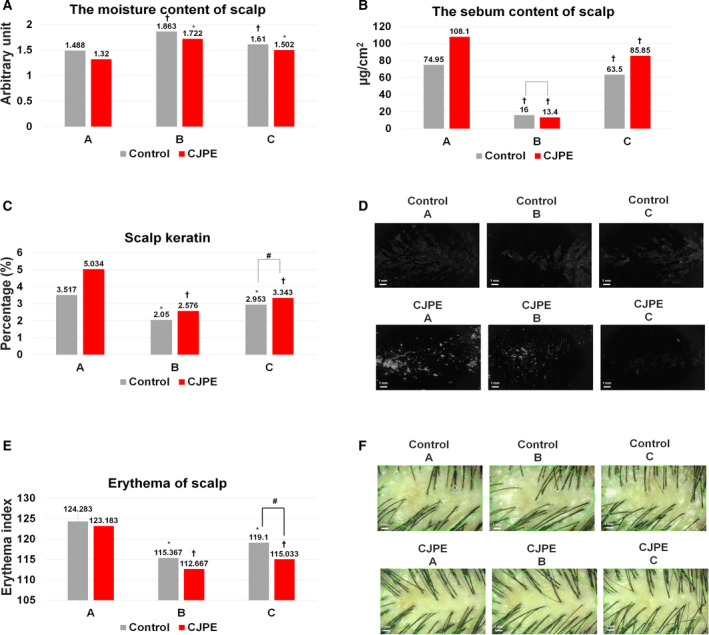
*In vivo* clinical tests of CJPE for scalp moisture content, sebum content, keratin, and erythema. The scalp moisture content (A), sebum content (B), keratin (C), and redness (E) were measured at Time Points A, B, and C. Control group (*N* = 20) and CJPE treatment group (*N* = 20). Change rate (%) = [(After product use – Before product use) / Before product use] × 100. Following statistical tests were used. Comparison within groups *: *P* < 0.05 by paired‐samples *t*‐test, ^†^: *P* < 0.05 by Wilcoxon signed‐rank test. Comparison between groups ^#^: *P* < 0.05 by independent *t*‐test. The *N* value (number of biologically independent replicates) was 20. The error bars represent the SD of a data set. (D) Representative images of scalp keratin after treatment with CJPE and sterile water (control) at three different time points. Images were taken by a Visioscan VC98. The white colored scale bar indicates 1 mm. (F) Representative images of scalp after treatment with CJPE and sterile water (control) at three different time points. The white colored scale bar indicates 1 mm. Images were taken by a folliscope 4.0.

We examined the effects of CJPE on hair follicle cell improvement *in vivo* by carrying out clinical tests with 42 adult female participants (21 using the CJPE product and 21 using the control product). Of the 42 participants, one only participated in the group using the control product, and one only participated in the group using the CJPE product. After participants used the test and control products, we measured the moisture content of the scalp in individual participants at three different time points: before (Time Point A), immediately after (Time Point B), and 1 week after product use (Time Point C) (Fig. 3A). The moisture content of the scalp in the group using the test product was significantly increased 34.169% at Time Point B and 14.997% at Time Point C as compared to Time Point A (*P* < 0.05). In the group using the control product, the moisture content was also significantly increased 27.273% at Time Point B and 9.651% at Time Point C as compared to Time Point A (*P* < 0.05). Moreover, there was no statistically significant difference between Time Points B and C for the group using the control product (*P* > 0.05), but the moisture content of the scalp for the group using the test product increased 6.896% at Time Point B and 5.346% at Time Point C as compared to the group using the control product.

Next, we measured the sebum content of the scalp in the two different groups (Fig. 3B). The sebum content of the scalp for the group using the test product was significantly decreased 82.118% at Time Point B and 27.768% at Time Point C as compared to Time Point A (*P* < 0.05). Similarly, that of the group using the control product was statistically significantly decreased 64.999% at Time Point B and 18.469% at Time Point C as compared to Time Point A (*P* < 0.05). We found that there was a statistically significant difference between the two groups at Time Point B (*P* < 0.05). However, there was no statistically significant difference at Time Point C (*P* > 0.05). The sebum content of the scalp in the group using the test product was decreased 9.299% as compared to the group using the control product.

We examined the scalp keratin improvement (Fig. 3C,D). The proportion of dead scalp keratin in the group using the test product was significantly decreased 45.503% at Time Point B and 34.160% at Time Point C as compared to Time Point A (*P* < 0.05). Moreover, that of the group using the control product was significantly decreased 39.949% at Time Point B and 15.292% at Time Point C as compared to Time Point A (*P* < 0.05). There was no statistically significant difference between the two groups at Time Point B (*P* > 0.05); however, the proportion of dead scalp keratin in the group using the test product was decreased 5.554% as compared to the group using the control product. Furthermore, there was a statistically significant difference between the two groups at Time Point C (*P* < 0.05).

We examined scalp redness improvement by measuring the erythema dose of the scalp area (Fig. 3E,F). The erythema of the scalp in the group using the test product was statistically significantly decreased 10.423% at Time Point B and 8.325% at Time Point C as compared to Time Point A (*P* < 0.05). Similarly, that of the group using the control product was statistically significantly decreased 7.993% at Time Point B and 4.787% at Time Point C (*P* < 0.05). In addition, there was no statistically significant difference between the two groups at Time Point B (*P* > 0.05); however, the erythema of the scalp in the CJPE group was decreased 2.430% as compared to the control group. There was a statistically significant difference between the two groups at Time Point C (*P* < 0.05).

### Transcriptome analysis of HFDPC in response to CJPE

**Fig. 4:**
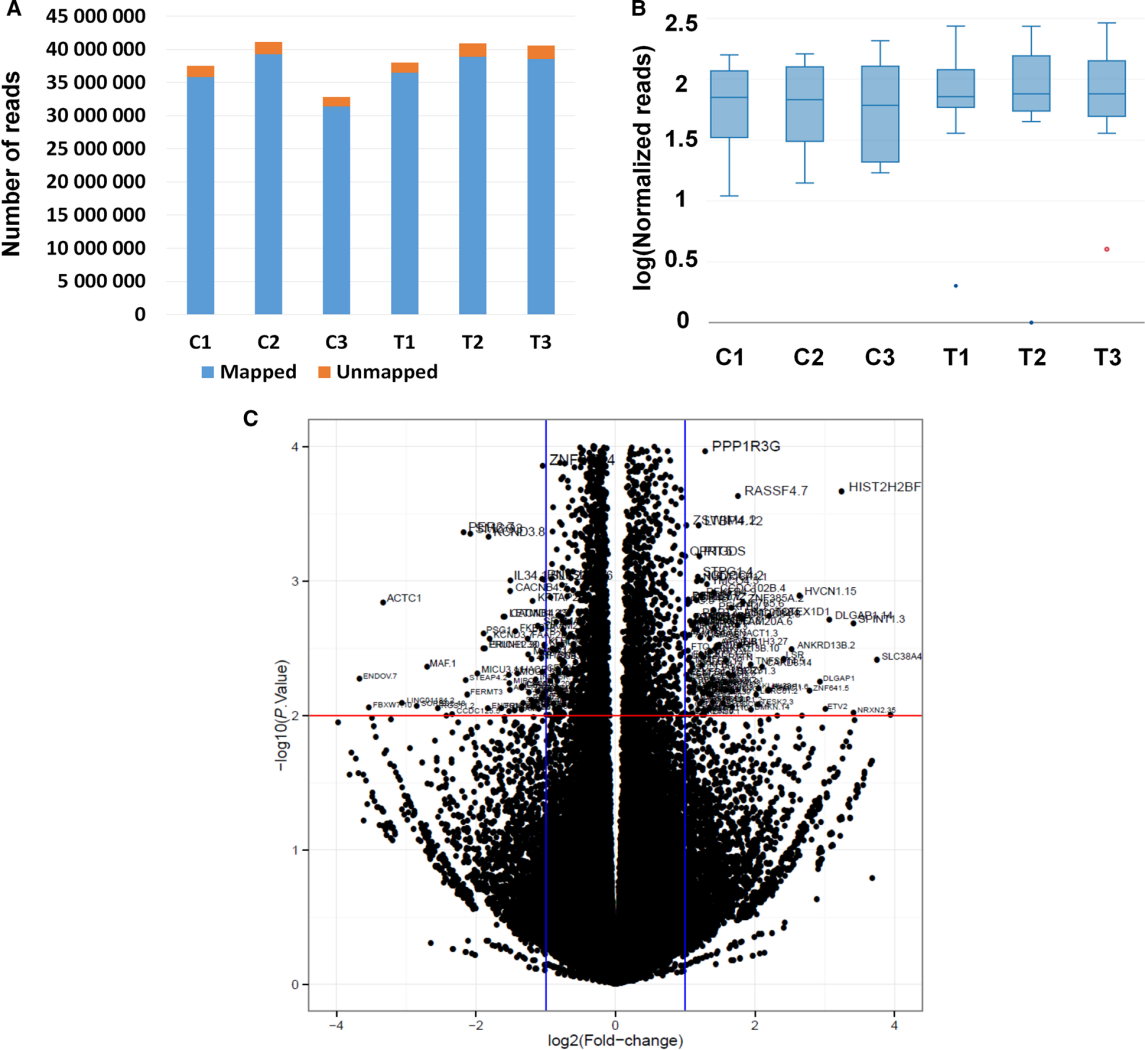
Mapping results, distribution of normalized reads, and visualization of DEGs. (A) Proportion of mapped (blue color) and unmapped (orange color) reads on human reference transcriptome. (B) Boxplot showing distribution of normalized reads in each library. (C) Volcano plot displaying distribution of log_10_ (*P*‐value) and log_2_ (FC) for all expressed genes. *P*‐value and FC indicate *P*‐value and fold change, respectively. DEGs are indicated with respective gene names.

Several previous studies have demonstrated positive effects of CJPE for medical and cosmetic agents. However, the detailed gene expression network caused by CJPE has not been elucidated. In this study, we carried out RNA‐seq to identify human DEGs in response to CJPE. First, we prepared two independent conditions, treatment (HFDPC treated with CJPE) and control (HFDPC treated with sterile water), with three biological replicates. After extracting total RNA from the samples, we generated six different mRNA libraries for RNA‐seq, which were paired‐end sequenced by the NovaSeq 6000 system. We obtained a total of 23 GB data (230 675 316 reads) from the six libraries (Table 1) and mapped the raw sequence reads from each library on the human reference transcriptome (GRCh38). The C3 library showed the lowest number of reads among the six libraries (Fig. 4A). The proportion of mapped reads in each library ranged from 95.08% to 95.83%. The number of mapped reads for each transcript was used for normalization using DESeq2 implemented in GENAVi (https://junkdnalab.shinyapps.io/GENAVi/). The three libraries from the control condition showed a wide range of distribution as compared to those from the treatment condition (Fig. 4B). The number of normalized reads in the treatment condition was slightly higher than that in the control condition (Fig. 4B). Based on mapped reads, 115 413 (72.13%) out of 159 998 transcripts were expressed in the three control samples, whereas 115 338 (72.08%) transcripts were expressed in the three treated samples.

**Table 1:** Summary of raw sequence data and accession numbers. All raw sequences were deposited in NCBI's SRA database with respective accession numbers under PRJNA631804.

Sample	Condition	Accession Nos.	Total reads bp	Total reads	GC (%)
C1	Control sample replicate 1	SRR11774231	3 787 086 910	37 495 910	50.38
C2	Control sample replicate 2	SRR11774230	4 146 588 734	41 055 334	50.38
C3	Control sample replicate 3	SRR11774229	3 311 885 950	32 790 950	50.26
T1	CJPE‐treated sample replicate 1	SRR11774228	3 836 298 756	37 983 156	50.92
T2	CJPE‐treated sample replicate 2	SRR11774227	4 125 599 318	40 847 518	50.76
T3	CJPE‐treated sample replicate 3	SRR11774226	4 090 747 248	40 502 448	50.82

### Identification of DEGs

In general, the effect of CJPE on the HFDPC transcriptome was strong, as can be seen in the scatterplot using log_2_ (fold change) and *P*‐values (Fig. 4C), and several genes were differentially expressed. Based on *P*‐values < 0.01 and log_2_ (fold changes) more than 1, we identified DEGs upon CJPE treatment as compared to the control. The number of upregulated genes (161 transcripts) was higher than that of downregulated genes (93 transcripts) (Table S1). The top 10 upregulated genes were *BANP, MYO15A, ANKRD20A19P, MYO5C, GLIPR1L1, KLHDC9, CCDC74B, DMGDH, NRL*, and *GNA14*, whereas the top 10 downregulated genes were *MYH6, CARD11, CCDC171, CCDC190, CCDC169, TTBK1, MECOM, RALGPS1, KCNA6*, and *DYSF* (Table 2). In addition, three long intergenic noncoding (LINC) RNAs were differentially expressed (Table S3). Of them, LINC00884 from chromosome 3 was highly upregulated, while LINC01184 from chromosome 5 and LINC01510 from chromosome 7 were highly downregulated.

**Table 2:** Top 20 representative DEGs upon CJPE treatment.

Gene name	Gene description	Log (FC)	*P*‐value
BANP	BTG3‐associated nuclear protein	5.324362932	0.000326
MYO15A	Myosin XVA	5.313778654	0.00048
ANKRD20A19P	Ankyrin repeat domain 20 family member A19	5.118412552	0.000855
MYO5C	Myosin VC	5.105945235	0.000826
GLIPR1L1	GLIPR1‐like 1	5.057070003	0.0012021
KLHDC9	Kelch domain‐containing 9	4.963476361	0.001335463
CCDC74B	Coiled‐coil domain‐containing 74B	4.958830135	0.00151921
DMGDH	Dimethylglycine dehydrogenase	4.918907191	0.00250749
NRL	Neural retina leucine zipper	4.895311815	0.001895238
GNA14	G protein subunit alpha 14	4.876743127	0.0022774
MYH6	Myosin heavy chain 6	−8.196653609	0.001533186
CARD11	Caspase recruitment Domain‐containing protein 11	−5.329062818	0.000377
CCDC171	Coiled‐coil domain‐containing 171	−5.13248846	0.001573234
CCDC190	Coiled‐coil domain‐containing 190	−5.12955154	0.001109091
CCDC169	Coiled‐coil domain‐containing 169	−4.956087058	0.001918571
TTBK1	Tau tubulin kinase 1	−4.79290426	0.004425356
MECOM	MDS1 and EVI1 complex locus protein EVI1	−4.79290426	0.004425356
RALGPS1	Ral GEF with PH domain and SH3‐binding motif 1	−4.783724102	0.003202812
KCNA6	Potassium voltage‐gated channel Subfamily A Member 6	−4.718459153	0.005555995
DYSF	Dysferlin	−4.69822663	0.005244367

Although some genes belonged to the same gene family, the expression of individual genes was different (Table S1). For example, out of eight DEGs containing the coiled‐coil domain, four genes (*CCDC102B, CCDC74B, CCDC78*, and *CCDC84*) were upregulated, while four genes (*CCDC125, CCDC169, CCDC171*, and *CCDC190*) were downregulated. Similarly, out of 13 genes in the zinc finger family, eight genes (*ZBTB20, ZDHHC23, ZNF385A, ZNF641, ZNF677, ZNF718, ZNF785*, and *ZSWIM4*) were upregulated, whereas five genes (*ZDHHC23, ZNF287, ZNF385D, ZNF644,* and *ZNF780B*) were downregulated. In the case of the solute carrier (SLC) gene family, the number of upregulated SLC genes (four) was much higher than the number of downregulated SLC genes (one). We also found that the expression of different transcripts from an identical gene was very similar (Table S1). For instance, six transcripts encoded by *PFKFB4* (encoding 6‐phosphofructo‐2‐kinase/fructose‐2,6‐biphosphatase 4) were all upregulated, and six transcripts encoded by *KLHL24* (encoding Kelch‐like family member 24) were all downregulated.

### Identification of enriched GO terms

**Fig. 5:**
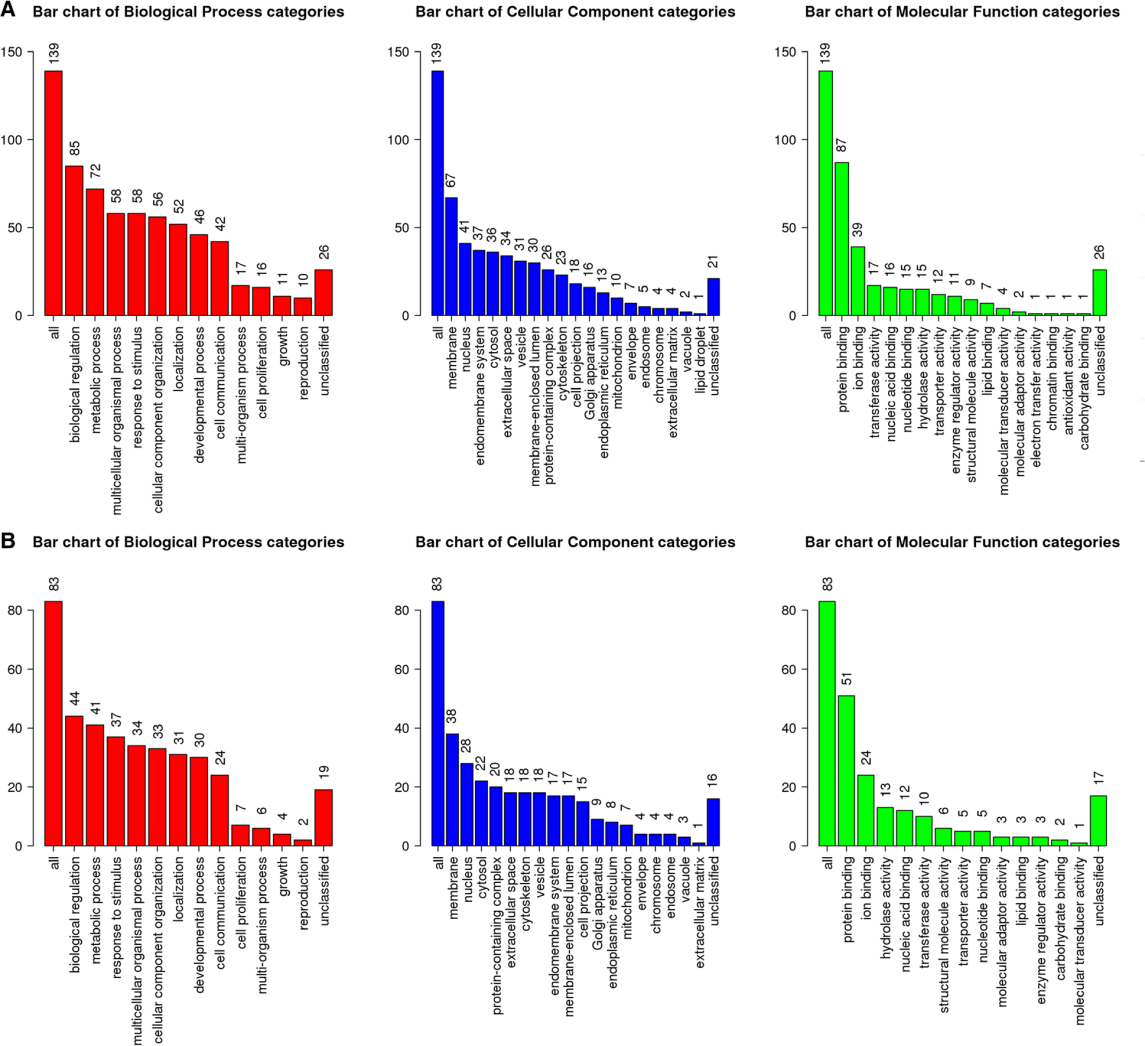
GO slim summary of identified DEGs. GO slim summary of upregulated genes (A) and downregulated genes (B). DEGs were divided into up‐ and downregulated gene groups. They were subjected to GO slim assignment using the WebGestalt tool. The three bar charts indicate the number of genes assigned to the GO slim terms according to BP (red bar chart), CC (blue bar chart), and MF (green bar chart) ontologies.

To reveal the functional distribution of identified DEGs, we conducted GO term enrichment analysis using the WebGestalt program. A total of 161 upregulated and 93 downregulated genes were assigned to GO terms according to BP, CC, and MF (Fig. 5). According to BP, the composition of GO terms of upregulated and downregulated genes was quite similar. For example, biological regulation (85 terms), metabolic process (72 terms), multicellular organismal process (58 terms), and response to stimulus (58 terms) were identified from upregulated genes (Fig. 5A), whereas biological regulation (44 terms), metabolic process (41 terms), response to stimulus (37 terms), and multicellular organismal process (34 terms) were identified from downregulated genes (Fig. 5B). According to CC, a large number of both up‐ and downregulated genes were assigned to the membrane and nucleus (Fig. 5A,B). However, the number of GO terms assigned to the endomembrane system was significantly higher in upregulated genes (37 terms) as compared to downregulated genes (17 terms). According to MF, a majority of genes were assigned to protein binding and ion binding in both gene groups (Fig. 5A,B). However, transferase activity was the third most important MF in upregulated genes (17 terms), whereas hydrolase activity was the third most important MF in downregulated genes (13 terms).

**Fig. 6:**
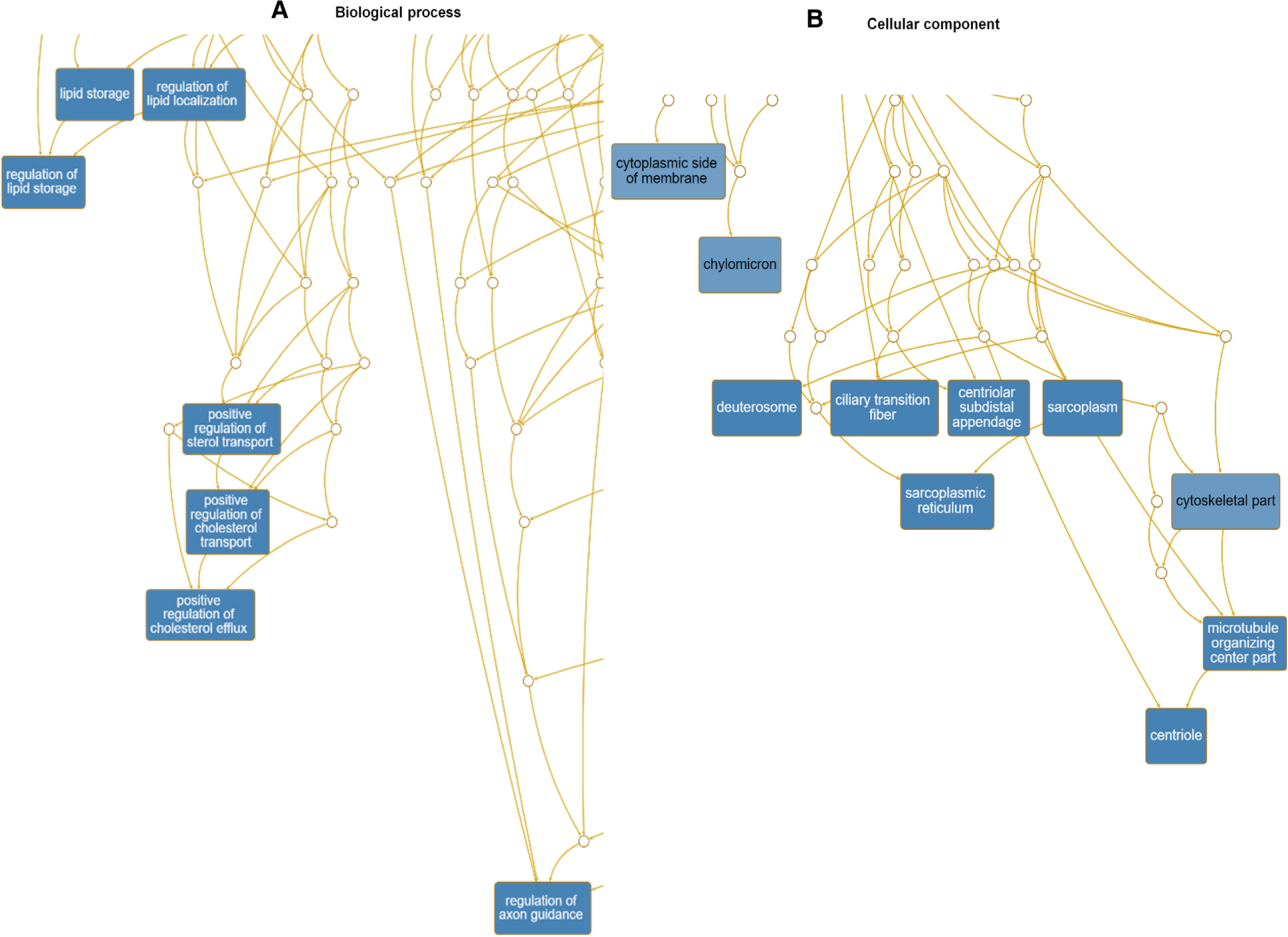
Hierarchical structure of identified enriched GO terms for upregulated human genes in response to CJPE. DAGs visualize hierarchical structure of identified enriched GO terms for upregulated genes upon CJPE treatment according to BP (A) and CC (B). Each GO term is indicated by a different box color based on *P*‐value (darker box colors indicate that the identified GO term is more significant). Detailed information on identified GO terms can be found in Table S2.

**Fig. 7:**
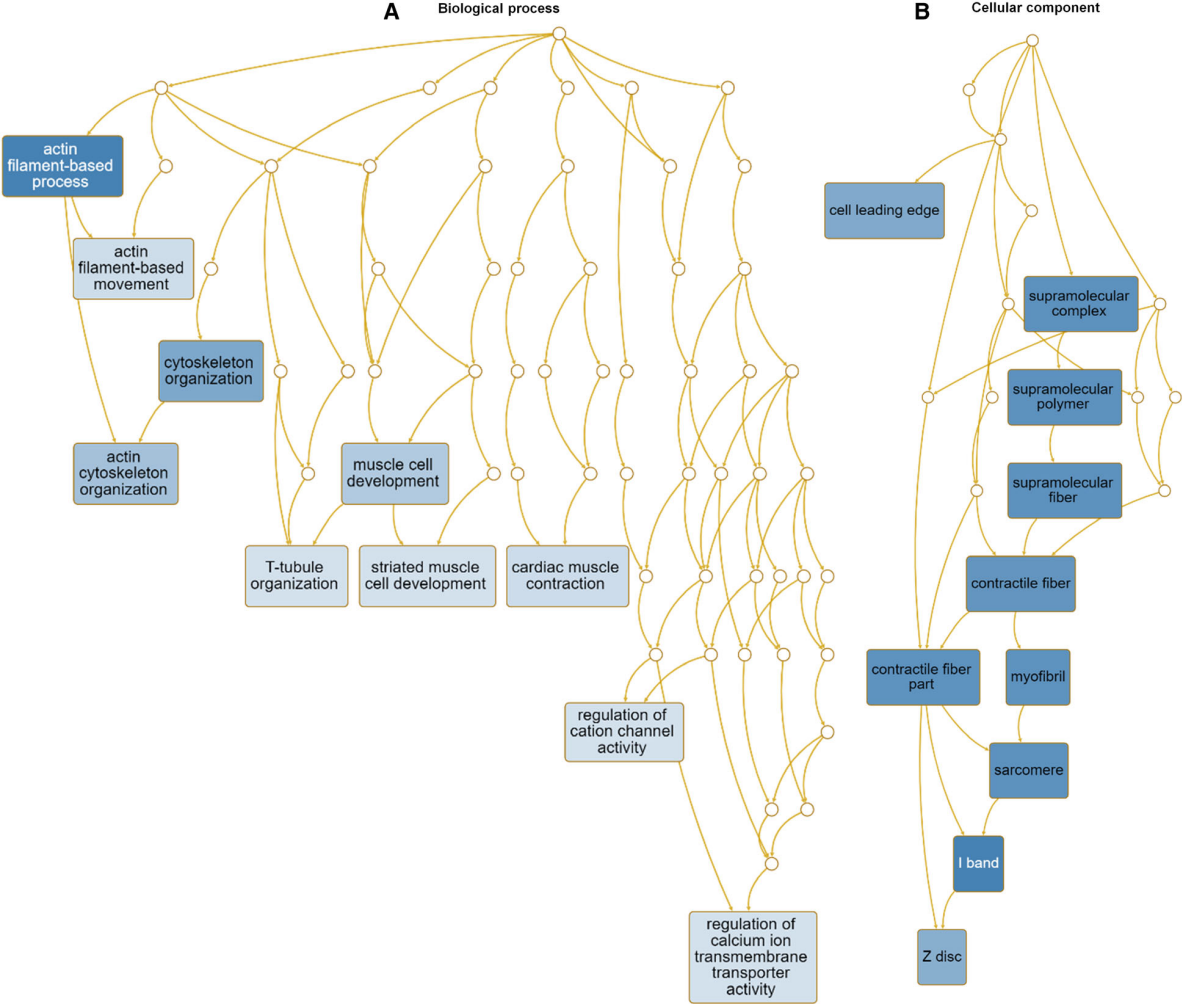
Hierarchical structure of identified enriched GO terms for downregulated human genes in response to CJPE. DAGs visualize hierarchical structure of identified enriched GO terms for upregulated genes upon CJPE treatment according to BP (A) and CC (B). Each GO term is indicated by a different box color based on *P*‐value (darker box colors indicate that the identified GO term is more significant). Detailed information on identified GO terms can be found in Table S3.

Next, we analyzed enriched GO terms using the WebGestalt program (http://www.webgestalt.org/). According to BP, GO terms associated with the regulation of lipid storage, positive regulation of cholesterol efflux, regulation of axon guidance, and positive regulation of the glycolytic process were enriched in upregulated genes (Table S2 and Fig. 6A). Many upregulated genes were located in the deuterostomes, sarcoplasmic reticula, and centrioles (Fig. 6B). According to MF, GO terms associated with signaling receptor binding and fructose‐2,6‐bisphosphate 2‐phosphatase activity were enriched (Fig. S1A). In the case of downregulated genes, GO terms associated with actin cytoskeleton organization and muscle development were highly enriched according to BP (Fig. 7A). Many downregulated genes were associated with supramolecular fibers, myofibrils, sarcomeres, and I bands (Fig. 7B). The enriched GO terms according to MF in downregulated genes were associated with structural constituents of the cytoskeleton, cytoskeletal protein binding, and actin filament binding (Fig. S1B).

In addition, we examined overrepresented pathways for upregulated genes against five databases: KEGG, Panther, Reactome, WikiPathways, and WikiPathways Cancer (Table S4). For upregulated genes, we identified axon guidance, fructose and mannose metabolism, parathyroid hormone synthesis, secretion, and action from the KEGG database. From the Panther database, we identified axon guidance mediated by Slit/Robo and the heterotrimeric G protein signaling pathway. From the Reactome database, plasma lipoprotein assembly, remodeling, and clearance and loss of Nlp from mitotic centrosomes were identified. From WikiPathways, we identified major receptors targeted by epinephrine and norepinephrine and nuclear receptors in lipid metabolism and toxicity, while the chemokine signaling pathway and the ATM signaling pathway were identified from the WikiPathways Cancer database.

For downregulated genes, we also identified several overrepresented functions from the five databases (Table S5). For example, cardiac muscle contraction, hypertrophic cardiomyopathy, and dilated cardiomyopathy from the KEGG database were overrepresented. From the Panther database, we identified cytoskeletal regulation by Rho GTPase and the circadian clock system, whereas muscle contraction and striated muscle contraction were identified from the Reactome database. Moreover, the striated muscle contraction pathway and cardiac progenitor differentiation were identified from the WikiPathways database, while phytochemical activity on NRF2 transcriptional activation and NRF2‐ARE regulation were identified from the WikiPathways Cancer database.

## Discussion

Scalp hair, consisting of keratin, plays important roles in protection from external pollutants and stresses as well as social and sexual interactions [31]. The hair growth cycle can be divided into three major phases, anagen, catagen, and telogen [31]. Several internal and external factors, such as stresses, pollutants, and hormones, negatively regulate the normal hair growth cycle, resulting in hair loss [32]. Hair loss diseases, including alopecia areata, alopecia totalis, and alopecia universalis, are very common in both males and females in modern society, and they are regarded as cell‐mediated autoimmune diseases [33]. The hair growth cycle is usually regulated by hair follicles derived from hair follicle stem and progenitor cells [31]. Dermal papilla cells, specialized mesenchymal cells that are the main component of hair follicles [31], generate signals that regulate hair induction, growth, and cycling [34].

To date, a large number of therapeutic candidates associated with hair induction and proliferation as well as hair growth have been developed [35, 36]. In this study, we evaluated CJPE as a potential therapeutic agent regulating scalp improvement. The *in vitro* assay of the *AQP3* gene using a molecular marker for moisturization demonstrated that CJPE is a promising moisturizing agent. Interestingly, among the three different CJPE treatment concentrations, 1% CJPE showed the highest expression of the *AQP3* gene, suggesting that the proper concentration of CJPE is required for clinical and cosmetic applications. Expression analyses for three hair growth marker genes encoding *CTNNB1*, *KGF*, and *VEGF* in HFDPC showed that all three genes were significantly increased upon CJPE treatment as compared to the control regardless of CJPE concentration. Moreover, we tested the expression of other hair growth marker genes in HFDPC after treatment with minoxidil and CJPE. We found that the treatment of minoxidil only induced the expression of the *ALP* gene out of the three examined genes, while CJPE induced the expression of all three genes. This result suggests that the mechanisms of minoxidil and CJPE for hair growth promotion might differ. Next, we compared the results of RT‐PCR with those of RNA‐seq for the three hair growth marker genes. By RNA‐seq, there was no difference in the expression of *CTNNB1* between the CJPE treatment and control. However, the expression of *KGF* (log_2_FC = 0.207, *P*‐value = 8.58E‐06) and *VEGF* (log_2_FC = 0.333, *P*‐value = 0.001894633) was slightly increased. It is likely that the difference in the normalization method between RT‐PCR and RNA‐seq affects the expression ratios [37]. Nevertheless, the upregulation of the three markers for hair growth suggests that CJPE is a promising agent for scalp homeostasis.

Although the *in vitro* assays indicated the possible hair follicle growth promotion by CJPE, it is important to demonstrate CJPE's efficacy by clinical tests. Our clinical tests with 42 adult female participants demonstrated that the product containing 0.5% CJPE increased the moisture content of the scalp and decreased its sebum content, dead scalp keratin, and erythema. In addition, we did not observe any side effects of the product containing 0.5% CJPE, suggesting the possible cosmetic application of CJPE for scalp improvement.

To date, numerous studies have shown the effects of *C. japonica* extract as a therapeutic agent for medicine and cosmetics; however, the detailed gene regulatory mechanism by CJPE has not been elucidated. Here, we conducted a genome‐wide transcriptome analysis by RNA‐seq to identify DEGs in HFDPC in response to CJPE treatment. RNA‐seq identified many DEGs regulated by CJPE, indicating that CJPE participates in the expression of several genes.

Treatment of HFDPC with CJPE increased genes encoding *ABCG1*, *APOE*, *FTO*, *HILPDA*, and *NR1H3* associated with lipid storage (GO:0019915). In addition, it increased genes encoding *CHCHD10*, *PFKFB3*, *PFKFB4*, *POMC*, *PPP1R3G*, and *ZBTB20* involved in the regulation of the generation of precursor metabolites and energy (GO:0043467). Moreover, three genes encoding *ABCG1*, *APOE*, and *NR1H3* required for the positive regulation of cholesterol efflux (GO:0010875) were upregulated by CJPE. Of the genes upregulated by CJPE, *ABCG1* is a member of the ATP‐binding cassette gene family and plays a role in the efflux of cholesterol from cells to high‐density lipoprotein [38]. A previous study demonstrated the expression of the *ABCG1* gene in human keratinocytes and murine epidermis, and its expression was induced during keratinocyte differentiation [38]. It is noteworthy that cholesterol homeostasis is important for the proper function of keratinocytes and the epidermis, as it mediates permeability barrier functions, as described previously [38]. APOE (encoding apolipoprotein E) functions in lipid transport, immunoregulation, Alzheimer's disease, and cognitive function [39]. A recent study showed that ApoE−/− mice resulted in skin inflammation and hair discoloration/loss, indicating a possible functional role of APOE in hair follicle formation [40]. As shown above, our results were highly consistent with several previous studies suggesting that lipid metabolism and cholesterol homeostasis are linked to hair growth and hair disorders [41, 42]. For example, a previous study demonstrated that Insig‐1 and Insig‐2 are involved in cholesterol biosynthesis and that Insig‐double knockout mice resulted in hair growth defects [43]. Moreover, mutations in a cholesterol transporter are related to congenital hypertrichosis, and dyslipidemia is associated with androgenic alopecia [41]. Thus, we hypothesized that CJPE might enhance hair follicle growth by stimulating the expression of genes involved in lipid metabolism and cholesterol efflux.

Several hormones are involved in hair follicle biology. For instance, three upregulated genes, *ADCY4, PDE4B*, and *PTHLH,* are involved in parathyroid hormone synthesis, secretion, and action (hsa04928) according to the KEGG pathway. Previous studies have demonstrated that the parathyroid hormone‐related protein derived from skin epithelial cells binds to receptors in dermal papilla cells and functions in the development of hair follicles [44, 45]. Three upregulated genes, *CPT1C, PFKFB3*, and *PFKFB4*, are involved in the AMPK signaling pathway (hsa04152). A recent study demonstrated functional roles of *PFKFB3* for the induction of proliferation and inhibition of differentiation in epidermal keratinocytes [46]. It has been reported that small molecules, such as rapamycin, metformin, and α‐KG, induce anagen hair growth by modulating mTOR and AMPK signaling, suggesting the involvement of the AMPK signaling pathway in hair growth [47]. One of the upregulated genes, proopiomelanocortin (POMC), is the common precursor of two hormones, adrenocorticotropic hormone (ACTH) and α‐melanocyte‐stimulating hormone (alpha‐MSH). Both hormones function in the regulation of human skin pigmentation as well as the differentiation of human scalp hair follicle melanocytes (HFM) [48, 49]. In addition, the μ‐opiate receptor and POMC are expressed in HFM, and the endogenous opiate, β‐endorphin, is involved in the regulation of follicular pigmentation [49]. Mutations in human POMC result in red hair pigmentation, suggesting its role in hair pigmentation [50]. Two upregulated genes, *ADCY4* and *ADRA2A*, are known as major receptors targeted by epinephrine and norepinephrine (WP4589). A recent study showed that keratinocyte proliferation is regulated by the norepinephrine hormone to promote hair follicle growth [51]. Therefore, *ADRA2A* encoding adrenoceptor alpha 2A might be involved in hair follicle growth by interacting with norepinephrine.

Interestingly, four genes encoding *CXCL12*, *SEMA4A*, *SEMA6C*, and *ZSWIM4* required for the regulation of axon guidance (GO:1902667) were upregulated by CJPE. The axon is a nerve fiber and is important for nervous system development [52]. Of them, the *CXCL12* gene encodes C‐X‐C motif chemokine 12 (CXCL12), also known as stromal cell‐derived factor 1 [53]. CXCL12 is a chemokine protein, which is a family of small cytokines, and is involved in homeostatic and pathological conditions by interacting with its receptor CXC chemokine receptor 4 (CXCR4) [53]. A previous study demonstrated that CXCL12 and its receptor CXCR4 function to regulate the migration and positioning of melanoblasts during hair follicle formation and cycling in the mouse [54]. In addition, it has been reported that hair follicles produce chemokines that are induced by stress [55]. Interestingly, the fat mass and obesity‐associated (FTO) gene, which is a well‐known marker for obesity, is increased by CJPE treatment [56]. However, we are unaware of any possible role of FTO associated with hair growth, although a previous study showed the possible role of POMC in severe early‐onset obesity in humans [50].

GO terms for several of the downregulated genes are assigned as structural constituents of the cytoskeleton (GO:0008092). For example, some genes, including *ACTC1, ANK2, ANKRD1, ATXN3, DAAM2, FRMD5, INPP5K, IQGAP2, KCND3*, and *LCP1*, are involved in the actin filament‐based process (GO:0030029). In addition, many downregulated genes encode several CCs for contractile fibers, including myofibrils, sarcomeres, I bands, and Z disks. Furthermore, several genes are involved in muscle cell development (GO:0055001) (*ACTC1*, *ANK2*, *ANKRD1*, *DYSF*, *MYH6*, and *SORBS2*), cardiac muscle contraction (GO:0060048) (*ACTC1*, *ANK2*, *FKBP1B*, *KCND3*, and *MYH6*), and striated muscle cell development (GO:0055002) (*ACTC1*, *ANKRD1*, *DYSF*, *MYH6*, and *SORBS2*). Thus, it is clear that CJPE might downregulate genes involved in muscle structure and development. In particular, three genes, *ACTC1*, *CACNB4*, and *MYH6*, are involved in cardiac muscle contraction (hsa04260).

Of the downregulated genes, *ACTC1* encodes alpha‐cardiac actin involved in normal cardiac morphogenesis [57] and atrial septal defects [58]. *ANKRD1* encodes ankyrin repeat protein 1, which is a member of the ankyrin repeat protein family [59] and is a cytokine‐inducible gene in microvascular endothelial cells [60]. It is known that ANKRD1 interacts with the type III intermediate filament desmin and that the loss of desmin induces the expression of ANKRD1 in smooth muscle cells [59]. *MYH6* encodes alpha‐cardiac myosin heavy chain, and mutations in *MYH6* result in familial atrial septal defects [61] and congenital heart defects [62]. SH3 domain‐containing protein 2 (SORBS2) is a scaffolding protein and a component of the apical junction complex that interacts with actin and several other cytoskeletal proteins [63]. Microarray analyses revealed that SORBS2 and TLR3 can induce senescence in primary human fibroblasts and keratinocytes [64]. In addition, SORBS2 functions as a potential tumor suppressor in cervical carcinogenesis [65], and loss of *SORBS2* function in mice leads to impaired dendritic development and memory [66]. Dysferlin (DYSF, encoding dystrophy‐associated fer‐1 like protein) is associated with skeletal muscle repair, and a defect in DYSF results in limb‐girdle muscular dystrophy type 2B and distal myopathy [67]. As a result, CJPE might downregulate several genes associated with the structural constituents of the cytoskeleton, such as actin, as well as muscle structure and development, such as cardiac muscle contraction.

## Conclusions

In summary, we demonstrated CJPE as a promising agent for hair growth promotion by *in vitro* assays showing the upregulation of the expression of hair growth marker genes. Moreover, *in vivo* clinical tests with 42 adult female participants showed that the product containing 0.5% CJPE improved the scalp by increasing the moisture content of the scalp and decreasing its sebum content, dead scalp keratin, and erythema. Furthermore, RNA‐seq analysis revealed key genes in HFDPC associated with CJPE. Interestingly, genes associated with lipid metabolism and cholesterol efflux were upregulated. In addition, CJPE upregulated genes associated with several hormones, such as parathyroid, ACTH, alpha‐MSH, and norepinephrine, which are involved in hair follicle biology. Furthermore, some upregulated genes were associated with the regulation of axon guidance. By contrast, many genes downregulated by CJPE were associated with structural components of the cytoskeleton, such as actin. In addition, CJPE suppressed genes associated with muscle structure and development. Taken together, this study provides extensive evidence of CJPE as a therapeutic agent for scalp improvement and hair growth promotion. In particular, RNA‐seq revealed the gene expression network of HFDPC in response to CJPE treatment.

## Conflict of interest

The authors declare no conflict of interest.

## Author contributions

SHM, HHS, and JHL conceived and designed the experiments. HIK, SHP, SYK, JS, OHL, JM, and SJL performed the experiments. WKC, YHJ, and HC conducted the bioinformatic analysis. WKC, HIK, and SHM wrote the manuscript. HIK, SYK, HHS, JS, OKL, JM, SJL, and JHL participated in the sample collection and data interpretation. WKC, HIK, SYK, HHS, and SHM contributed with valuable discussions and revision of the manuscript. All authors have read and approved the manuscript.

## Supporting information


**Fig. S1.** Hierarchical structure of identified enriched GO terms for upregulated and downregulated human genes in response to CJPE according to molecular function (MF). DAGs visualize hierarchical structure of identified enriched GO terms for upregulated genes (A) and downregulated genes (B) upon CJPE treatment according to MF. Each GO term is indicated by a different box color based on *P*‐value (darker box colors indicate that the identified GO term is more significant).

**Table S1**. DEGs in HFDPC in response to CJPE treatment. DEGs were identified based on fold changes of more than 2 and *P*‐values of less than 0.01. Red‐ and green‐colored shells indicate upregulated and downregulated genes, respectively.
**Table S2.** Identified enriched GO terms according to BP, CC, and MF for upregulated genes. GO enrichment analysis for upregulated genes was conducted using WebGestalt (http://www.webgestalt.org/). Top 10 enriched GO terms are listed in each GO category.
**Table S3.** Identified enriched GO terms according to BP, CC, and MF for downregulated genes. GO enrichment analysis for downregulated genes was conducted using WebGestalt (http://www.webgestalt.org/). Top 10 enriched GO terms are listed in each GO category.
**Table S4.** Overrepresented pathways for upregulated genes. Upregulated genes were subjected to analysis to identify overrepresented pathways in five functional databases: KEGG, Panther, Reactome, WikiPathways, and WikiPathways Cancer. Only top 10 functions from each database are listed.
**Table S5.** Overrepresented pathways for downregulated genes. Downregulated genes were subjected to analysis to identify overrepresented pathways in five functional databases: KEGG, Panther, Reactome, WikiPathways, and WikiPathways Cancer. Only top 10 functions from each database are listed.

## Data Availability

All raw sequences were deposited in NCBI's SRA database with respective accession numbers under PRJNA631804.
