# Supplementary figures and images for: Gene expression profile of human follicle dermal papilla cells in response to Camellia japonica phytoplacenta extract
Source: FEBS Open Bio. 2021 Feb 14;11(3):633–51. doi: 10.1002/2211-5463.13076 (PMC7931240; doi:10.1002/2211-5463.13076)

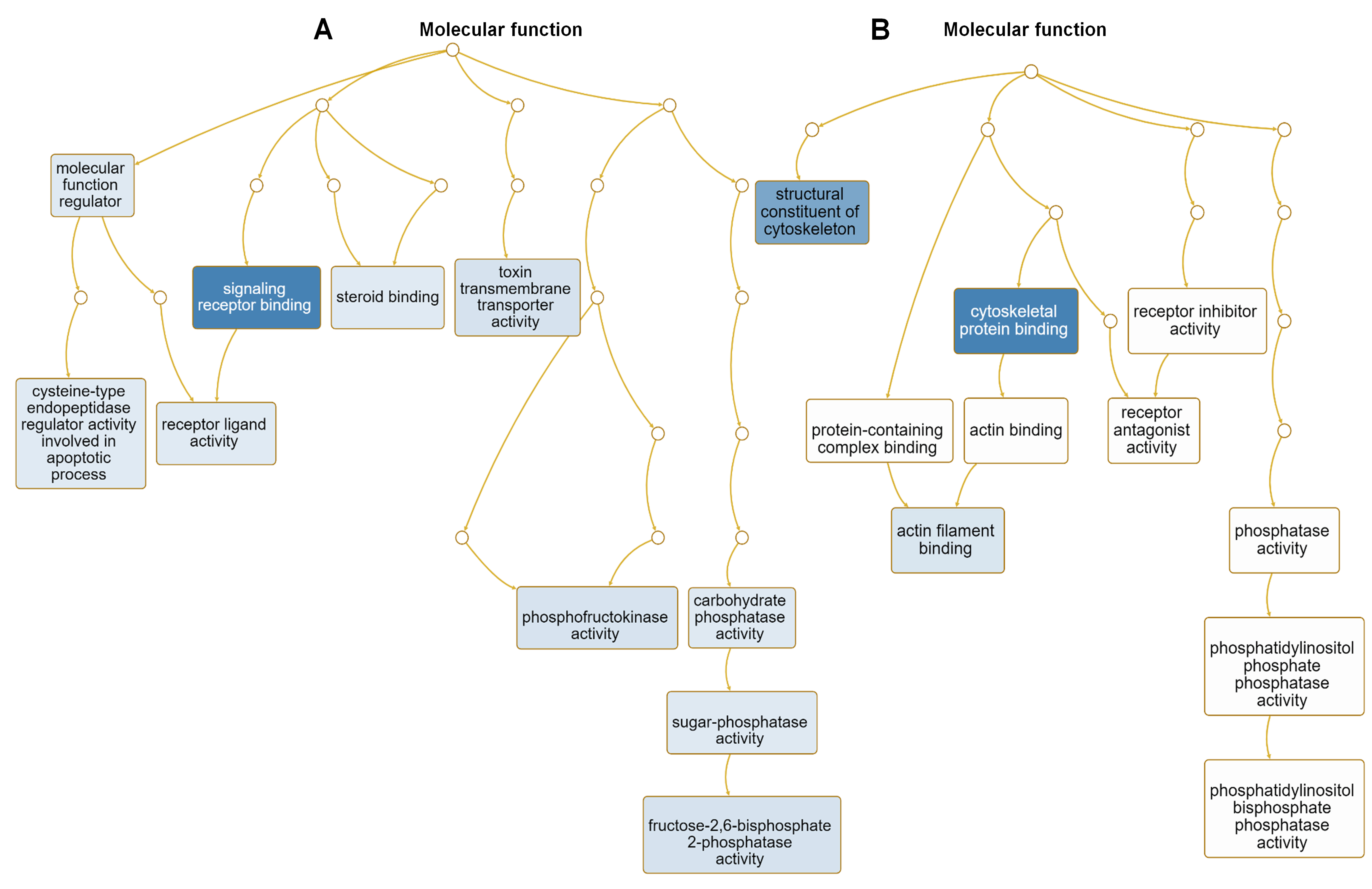

Supplement: Supplementary file 1 — Fig. S1. Hierarchical structure of identified enriched GO terms for upregulated and downregulated human genes in response to CJPE according to molecular function (MF). DAGs visualize hierarchical structure of identified enriched GO terms for upregulated genes (A) and downregulated genes (B) upon CJPE treatment according to MF. Each GO term is indicated by a different box color based on P‐value (darker box colors indicate that the identified GO term is more significant). [file FEB4-11-633-s001.jpg]
